# Rapid detection of West Nile and Dengue viruses from mosquito saliva by loop-mediated isothermal amplification and displaced probes

**DOI:** 10.1371/journal.pone.0298805

**Published:** 2024-02-23

**Authors:** Dongmin Kim, Terry J. DeBriere, Bradley H. Eastmond, Abdullah A. Alomar, Ozlem Yaren, Jacquelyn McCarter, Kevin M. Bradley, Steven A. Benner, Barry W. Alto, Nathan D. Burkett-Cadena

**Affiliations:** 1 Florida Medical Entomology Laboratory, University of Florida, Vero Beach, Florida, United States of America; 2 TrakitNow Inc., Columbia, South Carolina, United States of America; 3 Firebird Biomolecular Sciences LLC, Alachua, Florida, United States of America; 4 Foundation for Applied Molecular Evolution, Alachua, Florida, United States of America; University of South Florida, UNITED STATES

## Abstract

Arthropod-borne viruses are major causes of human and animal disease, especially in endemic low- and middle-income countries. Mosquito-borne pathogen surveillance is essential for risk assessment and vector control responses. Sentinel chicken serosurveillance (antibody testing) and mosquito pool screening (by RT-qPCR or virus isolation) are currently used to monitor arbovirus transmission, however substantial time lags of seroconversion and/or laborious mosquito identification and RNA extraction steps sacrifice their early warning value. As a consequence, timely vector control responses are compromised. Here, we report on development of a rapid arbovirus detection system whereby adding sucrose to reagents of loop-mediated isothermal amplification with displaced probes (DP-LAMP) elicits infectious mosquitoes to feed directly upon the reagent mix and expectorate viruses into the reagents during feeding. We demonstrate that RNA from pathogenic arboviruses (West Nile and Dengue viruses) transmitted in the infectious mosquito saliva was detectable rapidly (within 45 minutes) without RNA extraction. Sucrose stabilized viral RNA at field temperatures for at least 48 hours, important for transition of this system to practical use. After thermal treatment, the DP-LAMP could be reliably visualized by a simple optical image sensor to distinguish between positive and negative samples based on fluorescence intensity. Field application of this technology could fundamentally change conventional arbovirus surveillance methods by eliminating laborious RNA extraction steps, permitting arbovirus monitoring from additional sites, and substantially reducing time needed to detect circulating pathogens.

## Introduction

Mosquitoes are responsible for transmitting numerous and diverse arthropod-borne pathogens (arboviruses), which have major negative impacts on the health of humans and other animals worldwide. Each year, approximately three-quarters of a million people die from mosquito-borne pathogens [1], despite the large-scale efforts to eliminate or suppress the pathogens and their vectors. Mosquito-borne pathogen surveillance is a critical component of elimination and control programs, and it consists of the continuous and systematic collection, analysis, and interpretation of pathogen-related data from the field. This data is then used in the planning, implementation, and evaluation of public health practices [2, 3]. Active detection of arbovirus transmission serves as an important part of any early warning system. Under ideal conditions, surveillance allows for rapid response actions for targeted vector management, critical to protecting humans and animals against these pathogens [4, 5].

Diverse arbovirus surveillance strategies are available (S1 Fig), depending upon the pathogen, the vectors, the type of transmission (zoonotic versus anthroponotic), the geographic location, and the availability of technologies. During the Zika virus (ZIKV) epidemic, human case-based surveillance, involving IgM testing of human serum and cerebrospinal fluid [6], provided valuable information for vector control, despite its inability to distinguish between acute and prior infections. In Florida, USA, the sentinel chicken serosurveillance program is extensively used for early detection of zoonotic arboviruses [7, 8], such as West Nile virus (WNV), St. Louis encephalitis virus (SLEV), and eastern equine encephalitis virus (EEEV). This supports mosquito control districts in administering adulticide applications to interrupt transmission. The current approach allows for temporal and spatial resolution of past arbovirus infection events. While the sentinel chicken program is effective in detecting recent arbovirus transmission, it faces specific challenges. Firstly, there’s a time delay between chicken infection, seroconversion, and testing. Secondly, maintaining chicken flocks is costly and difficult. Thirdly, chickens are less effective in detecting viruses transmitted by mammal-biting *Aedes* species, such as ZIKV, dengue (DENV), and chikungunya (CHIKV) viruses [9]. Pool-screening of vectors by reverse transcription quantitative polymerase chain reaction (RT-qPCR) is highly sensitive and specific for arbovirus surveillance, providing information on spatial and temporal patterns, abundance, and identification of infected vector species. However, this technique requiring specialized equipment (thermal cycler) is labor-intensive/time-consuming (mosquito collection, mosquito identification and pooling, and RNA extraction), requires a modest molecular skillset, and a cold chain to preserve sample integrity [10]. Efficient techniques for arbovirus surveillance, utilizing both time and resources effectively, are essential to provide real-time notification of arbovirus circulation. This recommended approach will facilitate timely issuance of health advisories and the application of chemical interventions, thereby protecting both humans and nonhuman animals from potentially deadly mosquito-borne pathogens.

Exploiting sugar-feeding by trapped mosquitoes [11, 12] through the use of “honey cards” combines advantageous aspects of other surveillance techniques. As with sentinel chickens, the saliva of numerous mosquitoes is concentrated into a single sample per trap [11, 12]. Unlike sentinel chickens, which do not capture mosquitoes, trapped mosquitoes in honey card techniques can be assayed (pool-screened) when an arbovirus-positive honey card is detected [12], allowing for more detailed information (e.g., mosquito species infected, and number of infected mosquitoes). Honey cards have demonstrated feasibility for multiple targets, such as the Murray Valley encephalitis virus, EEEV, and WNV, aiding in species identification for control, similar to classical pool-screening using RT-qPCR. The feasibility of honey cards has been demonstrated for multiple targets (e.g., Murray Valley encephalitis virus, EEEV, and WNV). However, a field validation study in Florida [13] revealed a challenge where mosquitoes feeding at a relatively low rate in honey cards resulted in reduced arbovirus detection rates. It’s important to note that honey card techniques still involve time-consuming RNA extraction steps and require expensive equipment and supplies for RT-qPCR [10].

Reverse transcription loop-mediated isothermal amplification (RT-LAMP) is considered a cost-effective and sensitive alternative to RT-qPCR [14]. Advantages include amplification without RNA extraction from the sample [15], single temperature cycles [16, 17], sensitivity (<10 genetic copies of target nucleic acids), and rapidity of results (within 30 min). Results can be visually observed and identified by measuring fluorescence intensity [18], turbidity [19], or color changes [20] through the addition of molecular tags to the reaction mixture. This specificity is advantageous for testing relatively impure biological samples, making it suitable for on-site testing. This assay can be applied to a variety of pathogens, including viruses [10, 21], protozoan parasites (e.g., *Plasmodium*) [22], and bacteria (e.g., *Salmonella*, *Staphylococcus*, *Vibrio*) [23]. It is also useful for multiplexing and real-time monitoring, targeting different viruses in a single reaction at a time [21]. Recently, our group successfully detected viral RNA in RT-LAMP with displaced probes from quaternary ammonium functionalized paper by honey-induced mosquito salivation and showed potential fesibility of arbovirus stability and detection [24].

The sensitivity, simplicity, and rapidity of RT-LAMP are attractive for arbovirus surveillance systems. If arboviruses in mosquito saliva can be collected directly into RT-LAMP reagents rather than using the honey card technique, an easy-to-use screening platform could be established to monitor pathogen presence in an area, facilitating timely vector control response. Here, we combined the advantages of honey card and RT-LAMP with displaced probes (DP-LAMP) to assess the feasibility of rapid arbovirus detection. In iterative laboratory bioassays, our first objective is to determine the optimum sugar formulation for mosquito acceptance of sweetened DP-LAMP reagents. Our second objective is to validate the sensitivity and stability of arbovirus detection on sweetened DP-LAMP reagents over time. The third objective is to screen arboviruses directly from infectious mosquito saliva by DP-LAMP. Additionally, we aim to visualize an endpoint of DP-LAMP products using an optical image sensor. This work was performed with WNV and DENV-I, as well as their respective vectors, *Culex quinquefasciatus* Say and *Aedes aegypti* L., two of the most important mosquito-borne pathogen systems. This work integrates entomology, molecular biology, and engineering, with the future goal of developing a rapid arbovirus detection platform with low cost and adequate spatiotemporal coverage. This platform will streamline the capacity of mosquito control districts to perform timely arbovirus surveillance, resulting in an improved ability to protect humans and animals against deadly pathogens.

## Materials and methods

### Maintenance of mosquitoes

Laboratory colonies of both *Cx*. *quinquefasciatus* and *Ae*. *aegypti* were maintained under controlled environmental conditions (27.0 ± 0.5°C, 80.0 ± 5.0% RH, and a 14:10 (L:D) h photoregime) at the Florida Medical Entomology Laboratory (FMEL). Mosquito larvae were reared in enamel pans (24.8cm x 19.7 cm x 3.8 cm) containing 1.0 L of distilled water at low density (less than 500) for more homogeneity with respect to individual size. Mosquito larvae were fed an equal mixture of brewer’s yeast and lactalbumin or a diet of fish food, TetraMin^™^ (Tetra, VA, USA) on a standardized mosquito-rearing schedule [25]. Pupae were collected daily and transferred to 30 mL plastic cups containing distilled water. These cups were placed inside 24.0 cm x 24.0 cm mesh screen cages (BioQuip Products Inc., CA, USA) for adult eclosion. Adults were provided *ad libitum* 10% sucrose solution through/via cotton wool/balls in a 30 mL plastic cup placed inside each cage.

### Optimum sugar formulation for maximum mosquito feeding

We examined various sweeteners (carbohydrates) commonly found in natural nectar or commercial products to identify which sweetner elicited the highest feeding response in mosquitoes. These sweeteners included corn syrup (Lorann Oils, MI, USA), fructose (Modernist pantry, ME, USA), glucose (Modernist pantry, ME, USA), honey (Red Bunny Farms, PA, USA), sucralose (BulkSupplements, NV, USA), and sucrose (Publix, FL, USA). In no-choice assays, we placed 15 female mosquitoes (5–7 days old, laboratory colony) of either *Cx*. *quinquefasciatus* or *Ae*. *aegypti*, which had been starved for 24 hours, into 470 mL paperboard cups (Webstaurant Store, Lititz, PA, USA) covered with mesh netting. A cotton round (6 cm in diameter) was saturated with 5 mL of a 10% aqueous solution of each sweetener, dyed with 0.1 mL of blue food coloring (McCormick & Company, Inc., MD, USA) as a visual cue for sugar-feeding. The saturated cotton round was placed on an acrylic Petri dish inside each paperboard cup. Female mosquitoes were provided *ad libitum* access to the sweetener solutions over 24 hours in an environmental chamber. As a negative control, we included starved mosquitoes with no access to water or sugar. Five assay cups were tested for each sweetener and species. Subsequently, the mosquitoes were cold-anesthetized at -80°C, and their individual mass was measured using a microbalance (model Orion Cahn C‐33; Thermo Electron Corporation, MA, USA) with 1μg precision. The degree of sugar engorgement for each female was determined under a dissection microscope. Feeding extent was quantified as the mean mass after feeding and percent engorgement using a qualitative scale (0–4).

We assessed mosquito engorgement by testing various concentrations of sucrose and honey, under the assumption that increased feeding correlates with greater expectoration of saliva. Employing the same protocols as described earlier, we used starved mosquitoes (with no access to water or sugar) as a negative control, and provided cohorts of female mosquitoes with concentrations of 5%, 10%, 20%, and 40% of honey or sucrose, reflecting sugar concentrations commonly found in nectar [26]. Five assay cups (N = 15 / cup) of mosquitoes were tested with each sugar concentration and species.

To determine mosquito feeding acceptability on sweetened DP-LAMP buffer, either 410 μL of honey or sucrose solution (conc. 50.1%) was added in DP-LAMP reaction mixtures containing 50 μL of 10X isothermal amplification buffer (New England Biolabs, MA, USA), 30 μL of 100 mM MgSO_4_ (New England Biolabs, MA, USA), and 10 μL of blue food coloring for a 40% final concentration. The cotton round was saturated with 500 μL of sweetened DP-LAMP buffer and placed on an acrylic Petri dish inside the paperboard cup. Five assay cups (N = 15 / cup) were tested with sucrose or honey solution with or without DP-LAMP reagents. Starved females (no water or sugar) served as negative control and 40% solution of honey or sucrose served as a positive control.

### Sensitivity and stability of sweetened DP-LAMP over time

West Nile virus (Indian River County, FL, GenBank: DQ983578.1) or DENV-l (Key West, FL, GenBank: JQ675358.1) was propagated in tissue culture flasks (175 cm^2^) with confluent monolayers of Vero cells (Vero E6, ATCC CRL-1586). After 1 h of incubation at 37°C with a 5% CO_2_ atmosphere, 25 mL media: 199 media, 10% fetal bovine serum, 0.2% amphotericin B (Fungizone^®^), and 2% penicillin-streptomycin were added into tissue culture flasks by following previous methods [27]. Tissue culture flasks with virus were harvested after 3 (WNV) or 7 (DENV-I) days of incubation and diluted in a ten-fold serial dilution. Viral RNA from the diluted virus was extracted in accordance with the procedures by the QIAamp viral RNA mini kit (Qiagen, CA, USA) and was amplified with 20 μL reaction mixtures that contained the following components: 10.8 μL of PCR Master Mix reagents (Invitrogen, CA, USA), 2.2 μL of DEPC-treated water (Fisher BioReagents, PA, USA), 1 μL of 10 μM of each primer, and 5 μL of RNA template by RT-qPCR (Bio-Rad Laboratories, CA, USA) using methods described elsewhere [28, 29]. All viral RNA samples were stored at −80°C until required for experiments. Premixed DP-LAMP buffer consisted of 12.5 μL of WarmStart 2X MM (NEB, MA, USA), 2.5 μL of WNV or DENV-I 10 X DP-LAMP primers and probes (1.6 μM of FIP and BIP, 0.4 μM of LB for WNV or 0.5 μM of LB for DENV, 0.5 μM of LF for WNV or 0.4 μM of LF for DENV-I, 0.2 μM of F3, and B3, 0.1 μM of fluorescent FAM-3′ labeled probe and 0.15 μM of Iowa Black FQ-5′ labeled LB/LF probe), 0.5 μL of RNase Inhibitor (NEB, MA, USA), 0.5 μL of Antarctic UDG (NEB, MA, USA), 0.25 μL of ET SSB (NEB, MA, USA), and 0.2 μL of dUTP (Promega, WI, USA) [21, 24, 30]. An aliquot of 7.55 μL of aqueous sucrose was added to the pre-mixed DP-LAMP buffer to achieve specified concentrations, which included 0%, 5%, 10%, 20%, and 40%. The mixture was thoroughly homogenized and centrifuged for 3 s. An aliquot of 1.0 μL of viral RNA, containing 4.0 log10 PFU/mL of either WNV or DENV-I, was added as a template to the premixed DP-LAMP buffer. In each DP-LAMP buffer without sucrose, a positive control template containing 4.0 log_10_ PFU/mL of either WNV or DENV-I and a negative control (NTC) containing nuclease-free water were included. A positive control template containing 4.0 log_10_ PFU/mL of either 1.0 μL WNV or DENV-I and a negative control (NTC) containing nuclease-free water were included in each DP-LAMP buffer without sucrose. The total reaction volume was 25μL. Premixed DP-LAMP was performed in a thermocycler (Bio-Rad Laboratories, CA, USA) at 65°C for 30 s (60 cycles), 95°C for 300 s (1 cycle), and 37°C for 300 s (1 cycle). Fluorescence intensity from the DP-LAMP assays after thermal treatment was measured by relative fluorescence units (RFU). Primer and probe sequences are listed in S1 Table. To test the duration and stability of the DP-LAMP assay with the addition of sucrose under simulated field conditions, the premixed DP-LAMP buffers including 10 X DP-LAMP primers and probes were added to 40% aqueous sucrose. One set of premixed DP-LAMP buffers with 40% aqueous sucrose was used as a positive control template containing 4.0 log_10_ PFU/mL of WNV or non-template control containing nuclease-free water. Other sets of premixed DP-LAMP buffer with nuclease-free water (without sucrose) were used as a positive control template or non-template control. The premixed DP-LAMP buffer was stored in an environmental chamber (27.0 ± 0.5°C, 80.0 ± 5.0% RH) for up to 72h, and the DP-LAMP assays were performed in a thermocycler at 0, 8, 24, 48, and 72h. Fluorescence intensity from the DP-LAMP amplicons was measured by RFU.

### Detection of arboviruses from mosquito saliva by sweetened DP-LAMP

Five to seven-day-old females of Cx. *quinquefasciatus* or *Ae*. *aegypti* were individually infected by intrathoracic inoculation with 69 nL of Dulbecco’s Modified Eagle Medium (DMEM, Gibco, MT, USA) containing 5% EquaFetal (Atlanta Biologicals, GA, USA), 1% PenStrep (Gibco, MT, USA), and 1% L-Glutamine (Gibco, MT, USA) containing WNV or DENV-l in a concentration of 4.0 log_10_ PFU/mL using a programmable microinjector (Nanoject III, Drummond Scientific, PA, USA) after cold anesthesia for 3 min. The WNV and DENV-l titers utilized in this study fall within the concentration range observed in naturally or artificially infected mosquitoes [31–33]. This method permitted us us to obtain consistent numbers of infectious females with a low variablitiy in virus titer [34]. Control (sham) mosquitoes were also intrathoracically inoculated with 69 nL of only media (DMEM). All inoculated mosquitoes were transferred into a 470 mL paperboard cup (Webstaurant Store, Lititz, PA, USA) and covered with a double layer of mesh to prevent escape. The paperboard cups were placed with water-soaked cotton balls to provide moisture and held within incubators at 27.0 ± 0.5°C, 80.0 ± 5.0% RH, and 14:10 (L:D) h photoregime for a 9-day incubation period, which is expected to be sufficient time for dissemination and salivary gland infection for both viruses [34].

No-choice assays were performed by placing a single female mosquito of either WNV-infected *Cx*. *quinquefasciatus* or DENV-I-infected *Ae*. *aegypti*, starved for 24 h, into a 33 mL clear polystyrene plastic vial. A 24 μL sweetened premixed DP-LAMP buffer including primers and probes was applied into the cap of the assay tube inserted into the bottom of the plastic vial which mosquitoes permitted *ad libitum* access to sweetened DP-LAMP buffer and directly salivated virus-infected mosquito saliva into the assay tube. Once DP-LAMP buffer feeding was confirmed individually by observation (visualization of blue coloring in the mosquito crop), we screened the viruses from sweetened DP-LAMP buffer using DP-LAMP assay. Then, virus-infected mosquitoes were anesthetized using ice, and wings and legs were removed for saliva collection. The proboscis of each female was inserted into a microhematocrit capillary tube (Fisherbrand, Houston, TX, USA) containing 100 μl of mineral oil (Cargille, NJ, USA) and was left to salivate for at least 45 min at room temperature. The capillary tube contents containing mosquito saliva and virus were expelled into a polypropylene centrifuge tube with 300 ul of DMEM. The viral RNA extractions were performed using the QIAamp Viral RNA Kit to screen viruses by RT-qPCR. The results were compared with their sensitivity and specificity. Samples returning a cycle threshold (CT) value of ≤38 were considered putatively positive.

### Visualization of DP-LAMP products by an optical image sensor

A blue light transilluminator was designed and then manufactured using a 3D printer (Ender-3 S, CREALITY, China) to capture an image of the DP-LAMP amplification for arbovirus detection based on turbidity, bioluminescence, and target specific probes (S3 Fig). Two 475 nm led (XPE BBL-L1-0000-00301-SB01, New Energy Ltd, NC, USA) one at each end of the enclosure, generated the blue light. The transilluminator was placed in a 220 X 200 x 100 mm cardboard box with a rectangular hole in its lid. The box supported a cellphone camera that viewed the samples through an amber 570 nm filter (2422 amber acrylic sheet, ePlastics, San Diego, CA, USA) covering the hole. After thermal treatment, we captured the optical image of DP-LAMP products in a polypropylene centrifuge tube with or without sunlight exposure at various WNV titers (i.e., 2, 3, 4, 5 log_10_ PFU) over time points (i.e., 0, 15, 30, 60 min). OpenCV with Python was used to measure hue (H-value), saturation (S-value), and brightness (B-value) with a range from 0 (black) to 100 (white).

### Statistical analysis

Differences in mean mass change in mosquitoes were determined by analysis of variance (ANOVA) test followed by a post hoc analysis for pairwise multiple comparisons. Kruskal Wallis non-parametric tests for statistical significance were used for percent engorgement between treatment groups. All statistical procedures were conducted by JMP Statistics, Version 15.0 (SAS Institute Inc., Cary, NC, USA). Alpha was set at 0.05 for all statistical tests.

## Results and discussion

**Fig 1:**
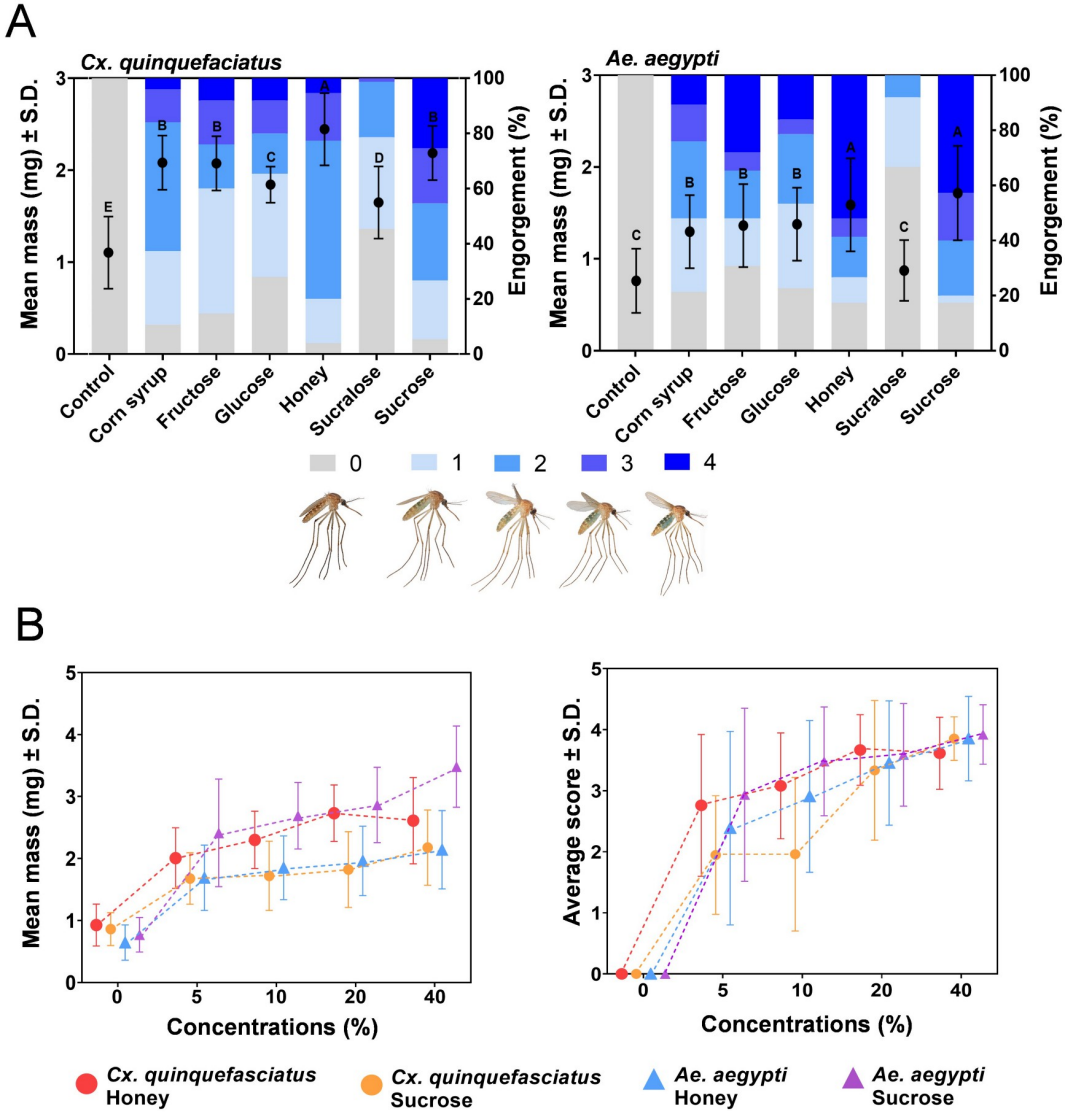
Palatability of various sweeteners for vector mosquitoes. Mean mass and engorgement of sweetener solutions for (A) *Cx*. *quinquefasciatus* and *Ae*. *aegypti*. Engorgement score (right y-axis) represents observable sugar solution (dyed blue) in the abdomen. Mean mass (left y-axis) recorded after 24-h access to treatments. Different letters indicate statistical significance (*p* < 0.05) by ANOVA with post hoc test. Error bars denote standard deviations. (B) Effect of honey and sucrose concentrations on mass and engorgement by *Cx*. *quinquefasciatus* and *Ae*. *aegypti*. In no-choice assays, females were provided access to honey or sucrose in 0%, 5%, 10%, 20%, or 40% aqueous solution for 24-h. Error bars denote standard deviations. The pictures of mosquitoes indicating the 0–4 ranking scale measuring sugar solution engorgement. Score 0: No visible sugar solution present; Score 1: Sugar solution visible only in the anterior and ventral parts; Score 2: Less than 30% engorged with sugar solution; Score 3: Approximately half full with sugar solution; Score 4: Nearly completely engorged with sugar solution.

**Fig 2:**
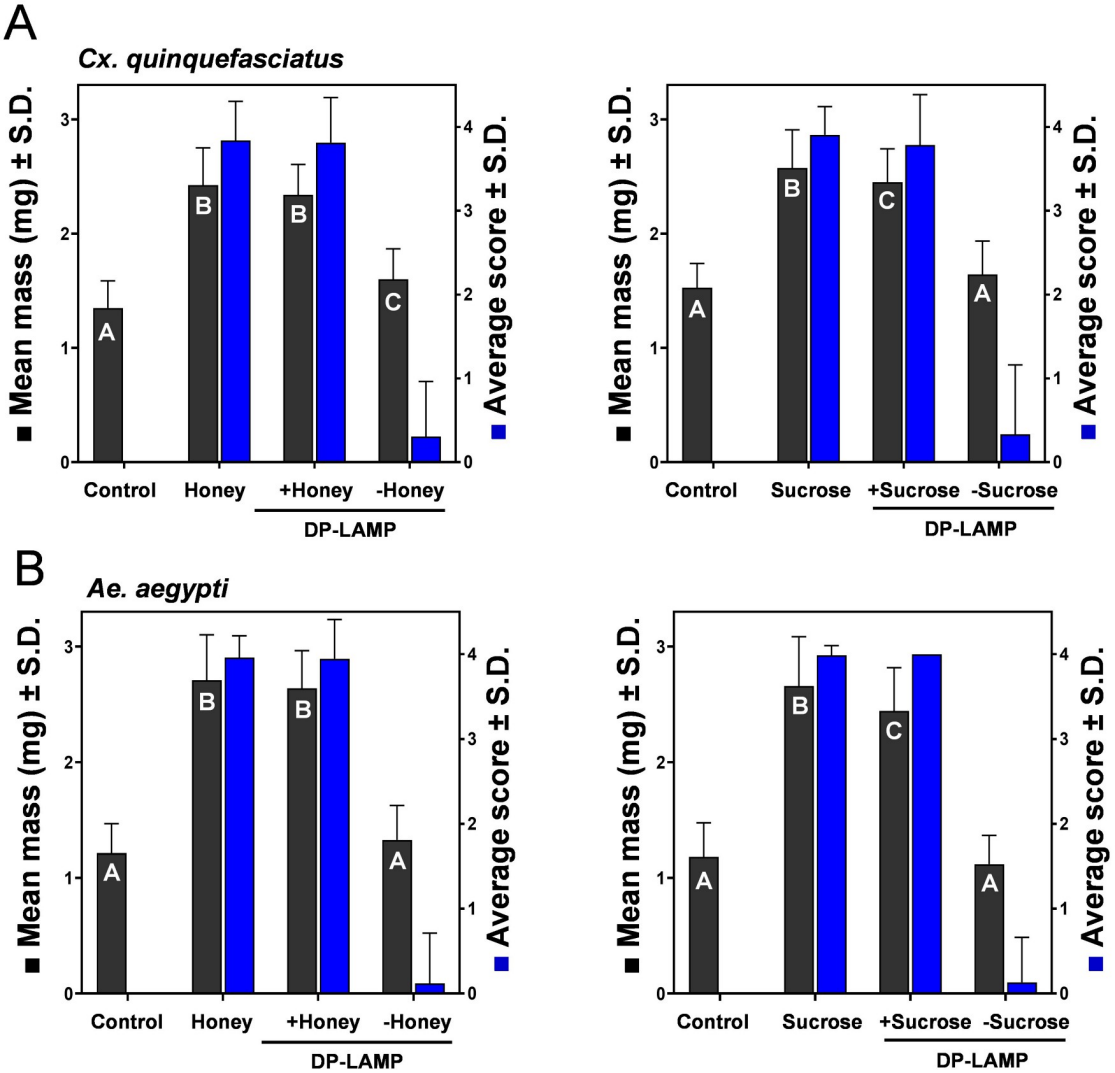
Palatability of sweetened DP-LAMP buffer for mosquitoes. Mean mass and engorgement of (A) *Cx*. *quinquefasciatus* and (B) *Ae*. *aegypti* on DP-LAMP buffer with and without honey (left panels) or sucrose (right panels). Engorgement score (right y-axis, blue bars) represents observable sugar solution (dyed blue) in the abdomen. Mean mass (left y-axis, black bars) recorded after 24-h access to treatments. In no-choice assays, females were provided access to DP-LAMP buffer, sweetened DP-LAMP (40% honey or sucrose), 40% sucrose or honey in aqueous solution, or control for 24-h. Different letters indicate statistical significance (*p* < 0.05) by ANOVA with post hoc test. Error bars denote standard deviations.

We determined an optimal sugar formulation as a critical parameter in the development of sweetened DP-LAMP assay to induce maximum feeding and associated salivation by measuring mosquito mass and scoring a volume of engorgement, from which we found greater engorgement in higher concentrations (20–40%) of honey and sucrose (both *p* <0.0001) (Fig 1A and 1B). Both male and female mosquitoes feed on a variety of sugar sources acquired from (extra) floral nectar [35], honeydew [36], fruits [37], plant tissues [38], and possibly vertebrate blood (i.e., glucose) [39]. Mosquitoes can differentiate between sugar diets through sugar-sensitive neurons located in sensilla on their proboscis and legs when given a selection of sugar sources [40]. This distinction is potentially associated with nutritional value and has significant effects on various aspects of their biological fitness, such as flight performance (e.g., swarming and host-seeking) [41], insemination rates [42], longevity [43], immune response [44], and vectorial capacity (resulting in reduced host blood meal size and frequency) [45]. The composition and concentration of floral nectar vary from plant species, age, and environmental conditions (e.g., temperature, and humidity) [25], but the common ranges of sugar concentrations found in nature are from 10 to 50% [25, 46]. Several studies showed that aroma volatiles increased with sucrose concentrations in dynamic headspace assays [47]. Also, the electrophysiological analysis identified that the higher sucrose concentration induced the maximal response of sugar-sensitive neurons in sensilla [48]. Although we did not determine the mosquito sugar preference by choice assays, our no-choice assays indicate that trapped females will successfully feed upon sweetened LAMP reagents. The tendency of feeding more on high concentrations of sugars in nature could be explained by evolutionary adaptation by two driving factors; energy and foraging costs [49] because higher sugar concentrations yield more energy and nutritional values for mosquito activities by reducing metabolic and predation costs. Mosquitoes were found to salivate more in highly concentrated nectars because it is too viscous to ingest [45], which is beneficial for arbovirus detection platform with sweetened DP-LAMP, assuming that more expectorated saliva positively relates to the amount of virus. We also found mosquitoes that fed on sweetened DP-LAMP buffer (honey or sucrose) were significantly higher in mass and engorgement (*p* <0.0001), compared to the only DP-LAMP buffer provided (Fig 2A and 2B). This observation is significant because higher engorgement levels are advantageous. We assume that greater engorgement leads to more saliva being deposited, resulting in a larger amount of virus available for testing. This, in turn, reduces the risk of false-negative results, ensuring an adequate quantity of viruses for detection using DP-LAMP.

**Fig 3:**
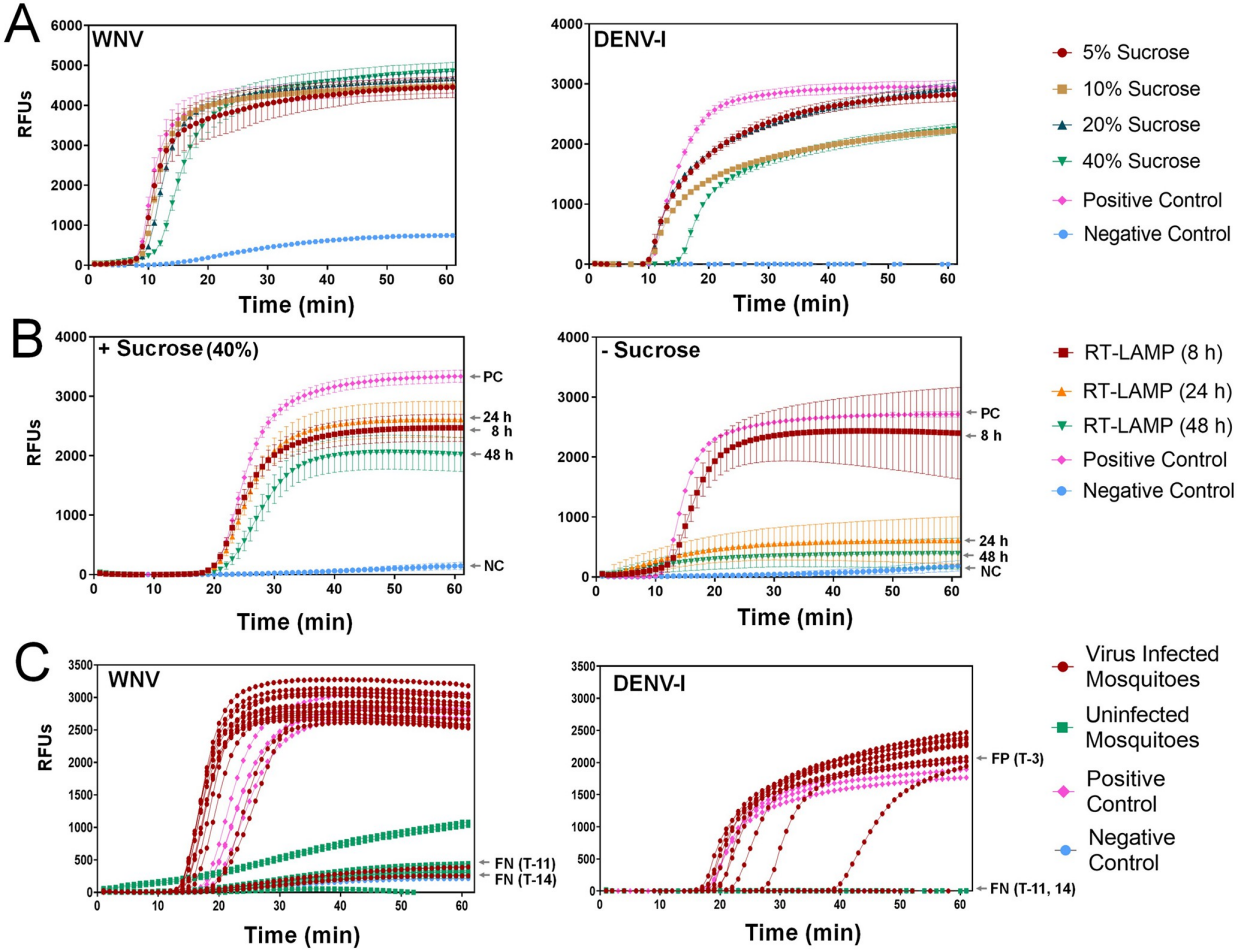
Sensitivity and specificity of sweetened DP-LAMP for arbovirus detection. (A) Effect of sugar concentration on detection of WNV and DENV-l using DP-LAMP. Relative fluorescence units (RFUs) corresponding to cycle number (time) of the West Nile virus (WNV) or Dengue-I virus (DENV-I) amplification on DP-LAMP assay mixed with various concentrations of sucrose solution. (B) Effect of sugar and incubation on stability and detection of WNV using DP-LAMP, incubated at 27.0 ± 0.5°C for 0, 8, 24, or 48 h. Sweeten DP-LAMP (+sucrose) indicates the reaction buffer mixed with 40% sucrose solution. DP-LAMP (-sucrose) indicates the reaction buffer mixed with nuclease-free water. (C) Detection of WNV and DENV-l directly from infectious mosquito saliva using sweetened DP-LAMP. Mosquitoes were infected with WNV 4.0 log_10_ PFU (*Cx*. *quinquefasciatus*) or DENV-I (*Ae*. *aegypti*) via microinjection followed by 9-day incubation. Arrows denote false positive (FP) or false negative (FN) samples: numbers correspond to S2 Table.

Through experimental screening for WNV and DENV-I, we determined that the DP-LAMP assay, when supplemented with various concentrations (0%-40%) of sucrose solution, effectively detects viral RNAs with high sensitivity and stability (Fig 3A). This capability enhances the feasibility of using DP-LAMP as a molecular detection tool for arboviruses. Our preliminary study showed that DP-LAMP assay could detect 10^−2^ PFU of WNV and 10^−3^ PFU of DENV-I, suitable for lower threshold values in arbovirus detection (S2 Fig). In a recent detailed study of comparison between DP-LAMP and RT-qPCR, a study by Burkhalter et al. [50] supports our results that DP-LAMP detected the same number of positives as RT-qPCR in laboratory assays and mosquito pools from the field and that DP-LAMP specificity was equivalent to that of RT-PCR. Most recently, a premixed DP-LAMP assay containing reagent/enzyme mixtures and coupled target-specific fluorescent tags detected multiple arboviruses, implemented with relatively impure biological samples (e.g., unprocessed urine) [21]. Importantly, viral nucleic material can be detected from even intact virus isolated in cell culture media without the need for an RNA isolation step, making it advantageous for field use and in a laboratory research setting. We also found that the sensitivity and specificity of sweetened DP-LAMP over various storage time periods (0-48h) under simulated field conditions (27.0 ± 0.5°C, 80.0 ± 5.0% RH up to 48 h) were significantly higher when compared without the addition of aqueous sucrose (Fig 3B). This result is supported by Lee et al. [51] and Shukla [52] indicated that sugar (e.g., sucrose) plays a role to preserve enzymatic activity without efficacy degradation and DP-LAMP reagents can be stable up to day 14 at room temperature. To date, RT-qPCR is a gold standard for sample analysis, but enzyme-based reagents should be maintained in a cold chain, presenting a limitation in the field surveillance and epidemiology for arbovirus detection. We expect DP-LAMP assay with the addition of aqueous sucrose will stabilize DP-LAMP efficacy to maintain functionality and stability for an extended period when a full field deployment of the arbovirus detection platform under hot and humid weather conditions.

We validated the sensitivity and specificity of viral RNA detection on sweetened DP-LAMP reagents by allowing infectious mosquito females to feed. Subsequently, we confirmed the results by employing RT-qPCR with saliva collected from the same mosquitoes during the DP-LAMP assay. The pairwise alignment of our results in DP-LAMP demonstrated that the viral RNAs directly from mosquito saliva exhibited a specificity (positive or negative) of 92.0% for WNV and 85.0% for DENV-I, in agreement with RT-qPCR results (Fig 3C and S2 Table). The two false-negative amplifications of WNV and DENV-I by DP-LAMP assay which were positive by collection of saliva in capillary tubes and subsequent RT-qPCR were likely due to insufficient feeding and expectoration in the sweetened LAMP assay. It was not feasible to determine whether a female that probed the sweetened LAMP buffer was actually feeding or merely probing the fluid. The false-positive (N = 1) DENV-I DP-LAMP sample may be due to off-target amplicons [53] and/or displaced probes may have caused non-specific amplification leading to a false positive, particularly based on the fluorescent strand and turbidity-based detection that have been observed in LAMP assays [54]. Our DP-LAMP assay consistently yielded optimal results through the adjustment of dNTPs, primers, and Mg2+ concentrations, which are crucial for reducing false positives and negatives [55]. This adjustment is essential to avoid potential over- or underestimation of arbovirus frequency in a given area. The RT-qPCR assay on mosquito whole bodies may not accurately reflect the true transmission risk in an area. The reason is that it only measures whole-body infection rates, and for human transmission to occur, the arbovirus must propagate within the mosquito and disseminate to secondary organs, including the salivary glands before being capable of introduction into humans through infected mosquito bites during blood-feeding. Therefore, the DP-LAMP assay presented in this study simplifies and more accurately reflects transmission events in the field through measurements of infectious vectors. The current approach has the potential to be integrated into an automated arbovirus detection system within a mosquito trap. The process comprises the following steps: 1) capturing vector mosquitoes alive into a collection chamber, 2) offering sweetened DP-LAMP for mosquito feeding, 3) amplifying the viral genome using a heating block, 4) screening fluorescence intensity through light transilluminator and camera observation, and 5) transmitting the results to a web interface via a cellular modem.

We employed a blue light transilluminator to visualize the endpoint of DP-LAMP products under various conditions: (1) sunlight exposure; (2) a gradient of titers using 10-fold serial dilutions of viral RNA (i.e., 2, 3, 4, and 5 log_10_ PFU); and (3) different time points (i.e., 0, 15, 30, and 60 min). Our results showed that the DP-LAMP products with WNV templates (4 log_10_ PFU/mL), regardless of conditions after a 65°C thermal treatment, produced 19.3% brighter green fluorescence compared to negatives, resulting in the ability to distinguish between positive and negative samples (S3 and S4 Tables). Non-ionizing radiation (UV radiation) is known as a major limiting factor photobleaching of the excited fluorescence [56]. However, we confirmed that fluorescence generated by a strand displacing activity had remarkable photostability and was consistent up to 60 min. In addition, fluorescence could readily indicate the presence or absence of these arboviruses regardless of viral titers, although it was not feasible to distinguish variations in titer among positive samples. Recently, colorimetric detection of the DP-LAMP reaction has been proposed as a diagnostic tool for arbovirus alternative to fluorescence intensity and turbidity-based detection [21, 57]. This new technique allows positive and negative amplifications to be distinguished immediately based on pH-induced color changes (e.g., violet-negative sample to sky blue-positive sample) under natural light. One major drawback is that the lower viral loads decrease the overall sensitivity of colorimetric assays [58], making it difficult to distinguish between positive and negative samples. The conventional diagnostic method (e.g., RT-qPCR) for arbovirus consists of three major steps: collection and identification of mosquito specimens, isolation of total RNA, and detection of amplified viral genome by RT-qPCR [13]. The latter typically takes at least 2 h to confirm the result. This procedure also includes several requirements including cold chain management. Therefore, increased testing burden, particularly during the peak prevalence of mosquitoes, limits early detection of arbovirus transmission critical for planning and deploying mosquito control actions. The sensitivity, simplicity, and rapidity of our optimal image system (S3 Fig) are greatly advantageous for use in field deployment under hot and humid environments. Also, our low-cost pre-screening method under $10 without a complex optical system (e.g., spectrophotometer) provides an excellent option for arbovirus detection, especially in endemic low- and middle-income countries.

We show here that sweetened DP-LAMP assays (S4 Fig) were capable of inducing mosquito feeding and salivation, collecting arboviruses from infectious mosquito saliva, and detecting viral RNAs (WNV and DENV-I) with high sensitivity and specificity, enabling robust identification of positive samples based on fluorescence intensity. This study suggests that our simple and reliable method has the potential to be easily integrated into existing trapping devices, significantly reducing the time and effort required for disease surveillance, including sample processing, reaction steps, and result interpretation, all without the need for RNA extraction. Future work will involve developing sweetened DP-LAMP assays for additional arboviruses, such as CHIKV, yellow fever virus, and EEEV. It will also include field testing a prototype mosquito trap integrated with various components, including sweetened DP-LAMP, a heating block, light transilluminator, camera, and a cellular modem. This testing is crucial for assessing the usability and feasibility of an automated arbovirus detection platform. The exploitation of sugar-feeding via an arboviruses detection system from vector mosquitoes by DP-LAMP assay has the potential for augmenting or perhaps even replacing sentinel chickens for arbovirus surveillance if the techniques are implemented by vector control districts.

## Supporting information

S1 Fig Pros and cons of different methods used for arbovirus surveillance. (TIF)

S2 Fig Sensitivity of the DP-LAMP and RT-qPCR for detection of WNV (A) and DENV-I (B). (TIF)

S3 Fig Diagram of 3D printed blue light (470 nm) transilluminator to capture an endpoint image of the DP-LAMP products for virus detection. (TIF)

S4 Fig (A) DENV infectious mosquitoes feeding directly on sweetened DP-LAMP reagents. (B) Capillary tube assay for collecting mosquito saliva containing DENV.
(TIFF)

S1 Table DP-LAMP primer and probe sequences used in this study. The DP-LAMP primers and strand-displaceable probes for DENV-I were purchased from IDT [24]. The WNV primer set was developed based on multiple sequences, utilizing 85 full genome sequences of WNV Subtype 1a obtained from human hosts and retrieved from viprbrc.org.
(DOCX)

S2 Table Results of DP-LAMP and RT-qPCR for West Nile virus (WNV) and Dengue-I virus (DENV-I) detection from mosquito saliva. P = positive; N = negative. N/A = not applicable.
(DOCX)

S3 Table Effect of sunlight exposure on stability and visualization of DP-LAMP products. Tubes with reagents with WNV at various viral titers (i.e., 2, 3, 4, and 5 log_10_ PFU) were exposed to sunlight over time (i.e., 0, 15, 30, and 60 min) and then visualized using blue LED and orange filter. Tubes without virus (letter “N”) were included as negative controls. The letter “P” indicates tubes with WNV.
(DOCX)

S4 Table Colorimetric values, including hue (H-value), saturation (S-value), and brightness (B-value), at the endpoint of DP-LAMP products with or without sunlight exposure at various viral titers (i.e., 2, 3, 4, and 5 log_10_ PFU) over time points (i.e., 0, 15, 30, and 60 min) were quantified using blue LED and orange filter and OpenCV with Python. (DOCX)
