# Supplementary figures and images for: Rapid detection of West Nile and Dengue viruses from mosquito saliva by loop-mediated isothermal amplification and displaced probes
Source: PLoS One. 2024 Feb 23;19(2):e0298805. doi: 10.1371/journal.pone.0298805 (PMC10889885; doi:10.1371/journal.pone.0298805)

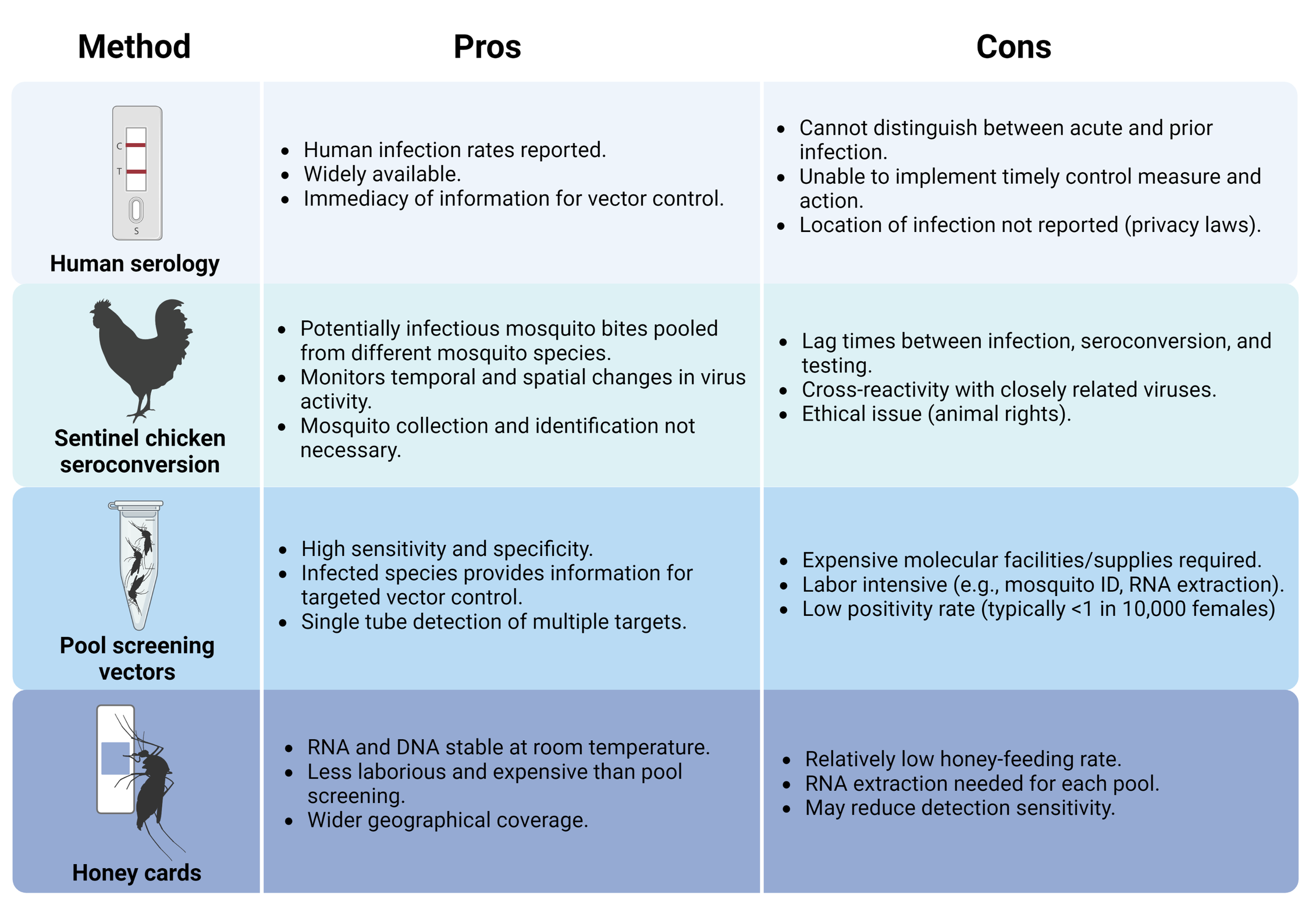

Supplement: S1 Fig — (TIF) [file pone.0298805.s001.tif]

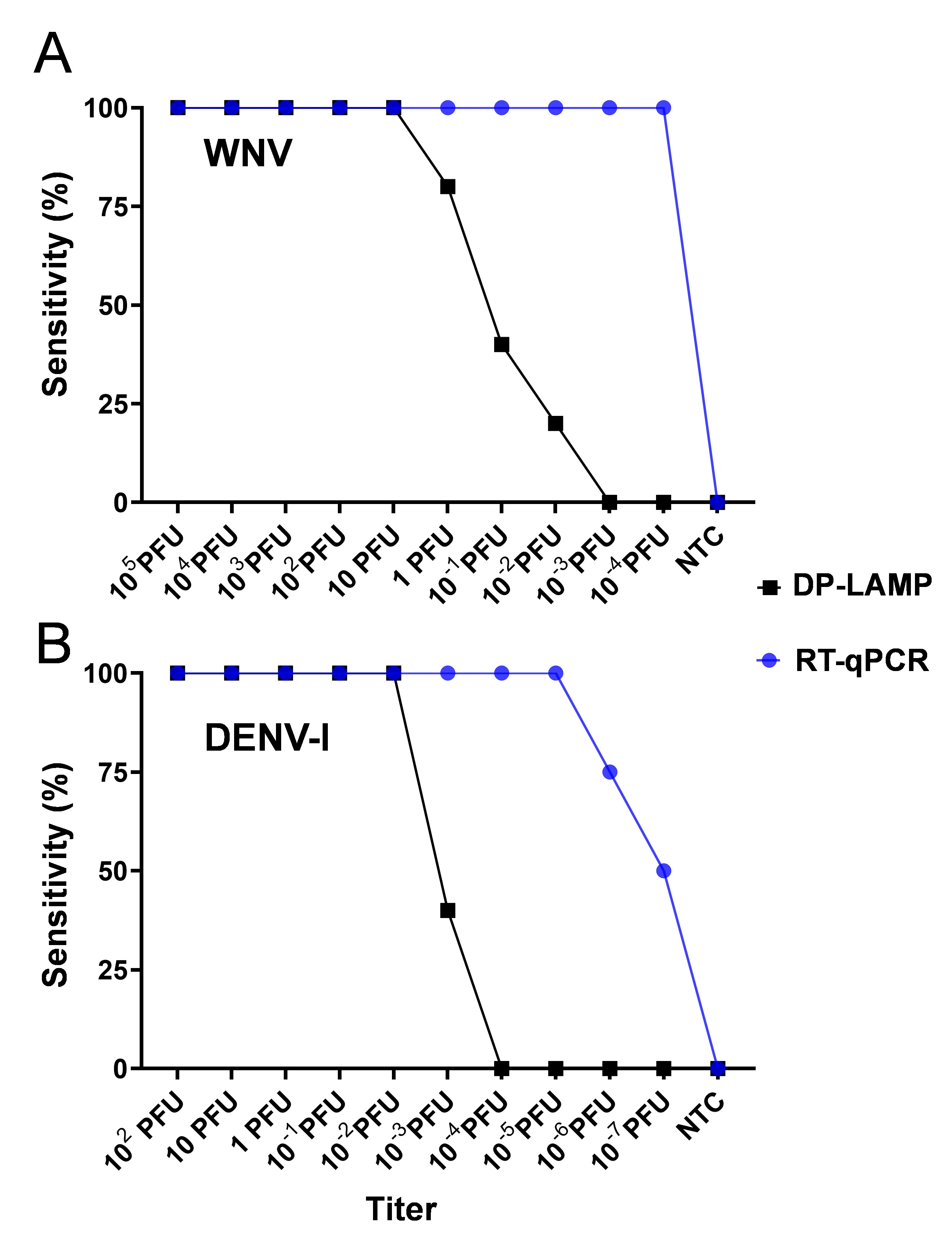

Supplement: S2 Fig — (TIF) [file pone.0298805.s002.tif]

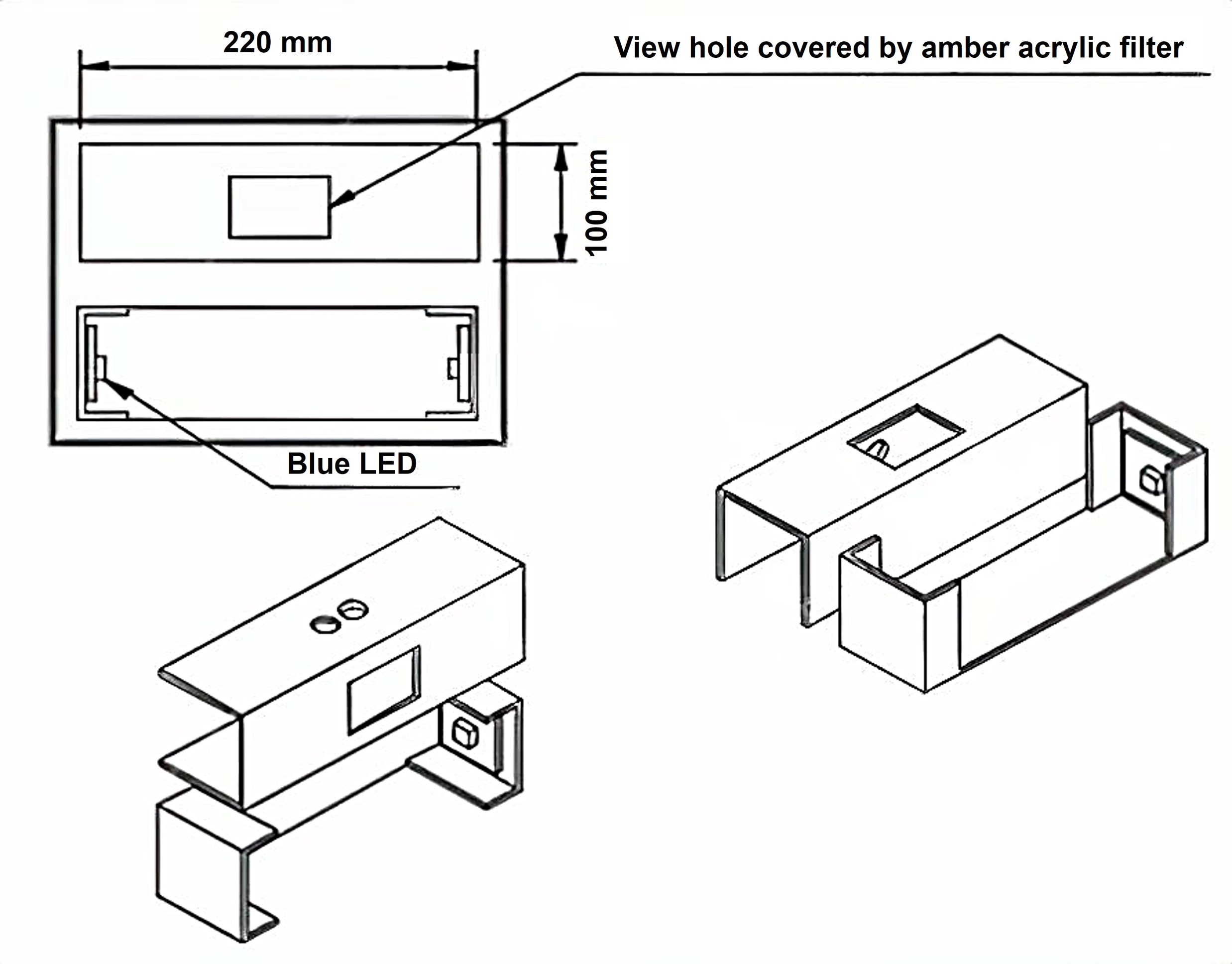

Supplement: S3 Fig — (TIF) [file pone.0298805.s003.tif]

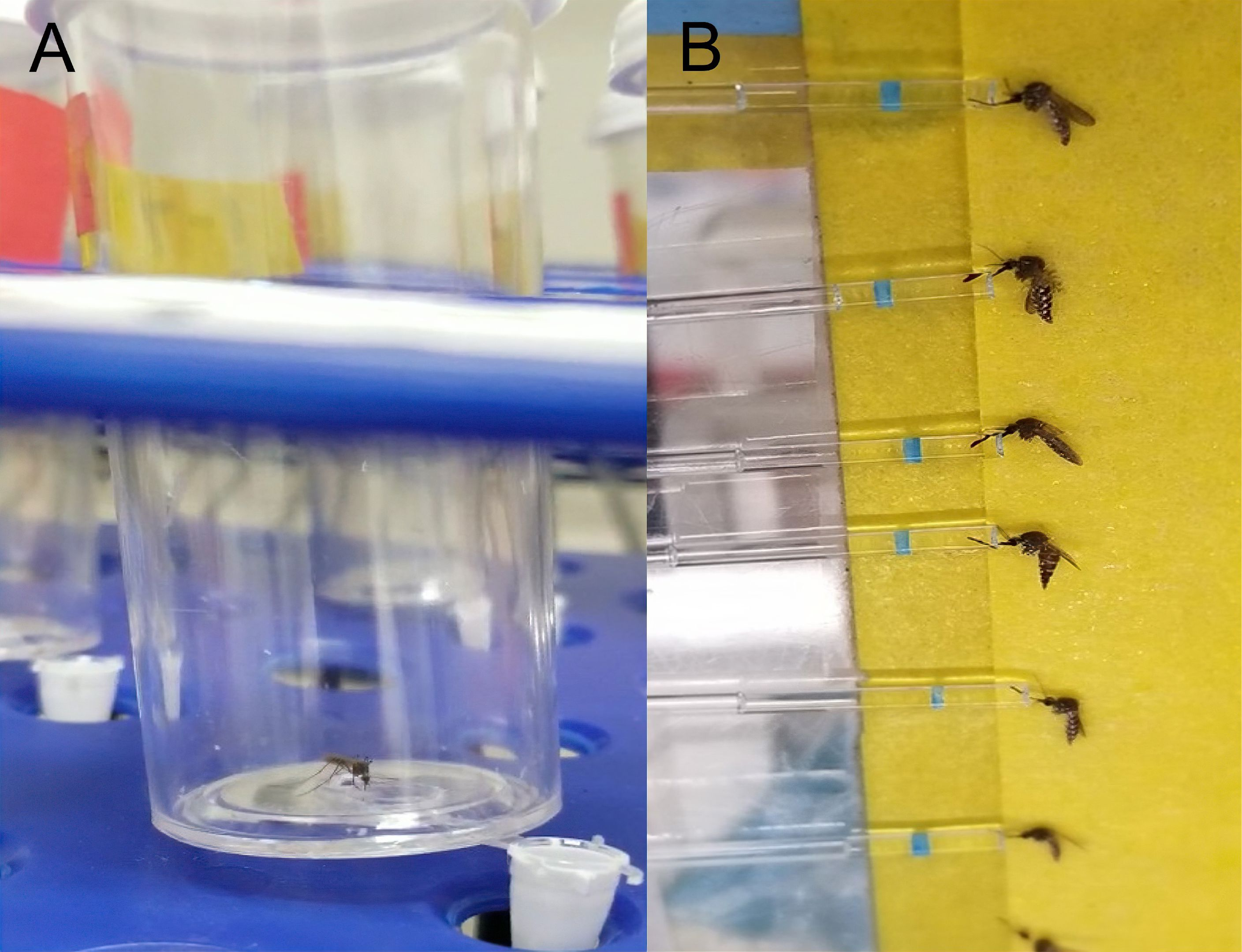

Supplement: S4 Fig — (A) DENV infectious mosquitoes feeding directly on sweetened DP-LAMP reagents. (B) Capillary tube assay for collecting mosquito saliva containing DENV. (TIFF) [file pone.0298805.s004.tiff]
