# Supplementary material for: Rapid detection of West Nile and Dengue viruses from mosquito saliva by loop-mediated isothermal amplification and displaced probes
Source: PLoS One. 2024 Feb 23;19(2):e0298805. doi: 10.1371/journal.pone.0298805 (PMC10889885; doi:10.1371/journal.pone.0298805)
Supplement: S1 Table — The DP-LAMP primers and strand-displaceable probes for DENV-I were purchased from IDT [24]. The WNV primer set was developed based on multiple sequences, utilizing 85 full genome sequences of WNV Subtype 1a obtained from human hosts and retrieved from viprbrc.org. (DOCX) [file pone.0298805.s005.docx]

**Table S1** DP-LAMP primer and probe sequences used in this study.

| **Target**  **virus** | **Name** | **Sequence (5’-3’)** |
| --- | --- | --- |
| DENV-I | D1_v2-1_F3 | CCTTCAAAAGGATGGAATG |
|  | D1_v2-1_B3 | TYAGGTCTCTCCTGTG |
|  | D1_v2-1_LB | CCTAGGCAARTCATATGCAC |
|  | D1_v2-1_LF | GTTCATCTTGGTTRCGGC |
|  | D1_v2-1_FIP | CCTTGTGATACTCTAGCCCTTTTTTGGGAAATAGTGGTGCC |
|  | D1_v2-1_BIP | GAGCCTGAGAGAAACTGCTTTTTGTACATCARCTGCCAC |
|  | D1v2-1 LF-tail13-5Q | ‘5IABkFQ’TCCCGGGCGAGAAAGATATTGTCCCTATGACGTCCGCTGC-GTTCATCTTGGTTRCGGC |
|  | Tail13-comp-3FAM | GCAGCGGACGTCATAGGGACAATATCTTTCTCGCCCGGGA‘36-FAM’ |
| WNV | WNV1a_2021-6_FIP | GACGARGACTCTCCGATGTTTTTGAGTGGATGTGTTYTACAG |
|  | WNV1a_2021-6_BIP | GCTGAGGTTGAAGAGCATAGTTTTTGGTGCARCCAGTCCTC |
|  | WNV1a_2021-6_LF | GTGTCACARCACTCAGAAGG |
|  | WNV1a_2021-6_LB | ATYCGGGTCCTTGAAATGG |
|  | WNV1a_2021-6_F3 | GTGCAAAGTTATGGATGG |
|  | WNV1a_2021-6_B3 | ACTTTYGGCATGTAGGG |
|  | WNV1a-2021-6-LB-tail2s-IBFQ | ‘5IABkFQ’TGTGTCTGAGGCTACGACCGTTTACC-ATYCGGGTCCTTGAAATGG |
|  | tail2s-26_Comp3FAM | GGTAAACGGTCGTAGCCTCAGACACA‘36-FAM’ |
