# Supplementary material for: Rapid detection of West Nile and Dengue viruses from mosquito saliva by loop-mediated isothermal amplification and displaced probes
Source: PLoS One. 2024 Feb 23;19(2):e0298805. doi: 10.1371/journal.pone.0298805 (PMC10889885; doi:10.1371/journal.pone.0298805)
Supplement: S2 Table — P = positive; N = negative. N/A = not applicable. (DOCX) [file pone.0298805.s006.docx]

| **Table S2** Results of DP-LAMP and RT-qPCR for West Nile virus (WNV) and Dengue-I virus (DENV-I) detection from mosquito saliva. P=positive; N=negative. N/A=not applicable. |
| --- |
| \| Sample \| WNV \| \|  \| DENV-I \| \| \| --- \| --- \| --- \| --- \| --- \| --- \| \| DP-LAMP \| RT-qPCR \|  \| DP-LAMP \| RT-qPCR \| \| T^1^-1 \| P \| P \|  \| P \| P \| \| T-2 \| P \| P \|  \| P \| P \| \| T-3 \| P \| P \|  \| P \| N \| \| T-4 \| P \| P \|  \| P \| P \| \| T-5 \| P \| P \|  \| P \| P \| \| T-6 \| P \| P \|  \| N \| N \| \| T-7 \| P \| P \|  \| P \| P \| \| T-8 \| P \| P \|  \| P \| P \| \| T-9 \| P \| P \|  \| P \| P \| \| T-10 \| P \| P \|  \| P \| P \| \| T-11 \| N \| P \|  \| N \| P \| \| T-12 \| P \| P \|  \| N \| P \| \| T-13 \| P \| P \|  \| N/A \| N/A \| \| T-14 \| N \| P \|  \| N/A \| N/A \| \| T-15 \| P \| P \|  \| N/A \| N/A \| \| C^2^-1 \| N \| N \|  \| N \| N \| \| C-2 \| N \| N \|  \| N \| N \| \| C-3 \| N \| N \|  \| N \| N \| \| C-4 \| N \| N \|  \| N \| N \| \| C-5 \| N \| N \|  \| N \| N \| \| C-6 \| N \| N \|  \| N \| N \| \| C-7 \| N \| N \|  \| N \| N \| \| C-8 \| N \| N \|  \| N \| N \| \| C-9 \| N \| N \|  \| N/A \| N/A \| \| C-10 \| N \| N \|  \| N/A \| N/A \| \| PC^3^-1 \| P \| P \|  \| P \| P \| \| PC-2 \| P \| P \|  \| P \| P \| \| PC-3 \| P \| P \|  \| P \| P \| \| NC^4^-1 \| N \| N \|  \| N \| N \| \| NC-2 \| N \| N \|  \| N \| N \| \| NC-3 \| N \| N \|  \| N \| N \| \| False positive (%) \| 0.0 \|  \|  \| 5.0 \|  \| \| False negative (%) \| 8.0 \|  \|  \| 10.0 \|  \| |
| ^1^T (Treatment): mosquitoes inoculated with cell media containing WNV or DENV-I; ^2^C (Control): mosquitoes inoculated with only cell media; ^3^PC (positive control): template containing 4.0 log_10_ PFU WNV or DENV-I; ^4^NC (negative control): non-template containing nuclease-free water. |
