# Supplementary material for: Rapid detection of West Nile and Dengue viruses from mosquito saliva by loop-mediated isothermal amplification and displaced probes
Source: PLoS One. 2024 Feb 23;19(2):e0298805. doi: 10.1371/journal.pone.0298805 (PMC10889885; doi:10.1371/journal.pone.0298805)
Supplement: S3 Table — Tubes with reagents with WNV at various viral titers (i.e., 2, 3, 4, and 5 log10 PFU) were exposed to sunlight over time (i.e., 0, 15, 30, and 60 min) and then visualized using blue LED and orange filter. Tubes without virus (letter “N”) were included as negative controls. The letter “P” indicates tubes with WNV. (DOCX) [file pone.0298805.s007.docx]

| **Table S3** Effect of sunlight exposure on stability and visualization of DP-LAMP products. Tubes with reagents with WNV at various viral titers (i.e., 2, 3, 4, and 5 log10 PFU) were exposed to sunlight over time (i.e., 0, 15, 30, and 60 min) and then visualized using blue LED and orange filter. Tubes without virus (letter “N”) were included as negative controls. The letter “P” indicates tubes with WNV. |
| --- |
| \| Time (min) \| Sunlight condition \| \| \| \| \| \| \| \|  \| Dark condition \| \| \| \| \| \| \| \| \| --- \| --- \| --- \| --- \| --- \| --- \| --- \| --- \| --- \| --- \| --- \| --- \| --- \| --- \| --- \| --- \| --- \| --- \| \| WNV Plaque-forming unit (PFU) \| \| \| \| \| \| \| \|  \| WNV Plaque-forming unit (PFU) \| \| \| \| \| \| \| \| \| 10^5^ \| \| 10^4^ \| \| 10^3^ \| \| 10^2^ \| \|  \| 10^5^ \| \| 10^4^ \| \| 10^3^ \| \| 10^2^ \| \| \| N \| P \| N \| P \| N \| P \| N \| P \|  \| N \| P \| N \| P \| N \| P \| N \| P \| \| 0 \| **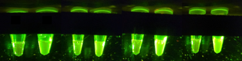** \| \| \| \| \| \| \| \|  \| **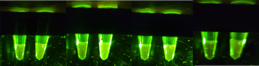** \| \| \| \| \| \| \| \| \| 15 \| **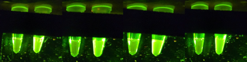** \| \| \| \| \| \| \| \|  \| **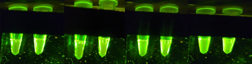** \| \| \| \| \| \| \| \| \| 30 \| **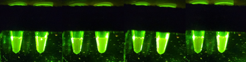** \| \| \| \| \| \| \| \|  \| **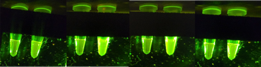** \| \| \| \| \| \| \| \| \| 60 \| **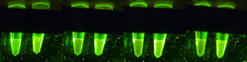** \| \| \| \| \| \| \| \|  \| **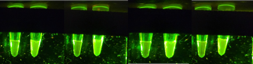** \| \| \| \| \| \| \| \| |
