# Supplementary material for: Rapid detection of West Nile and Dengue viruses from mosquito saliva by loop-mediated isothermal amplification and displaced probes
Source: PLoS One. 2024 Feb 23;19(2):e0298805. doi: 10.1371/journal.pone.0298805 (PMC10889885; doi:10.1371/journal.pone.0298805)
Supplement: S4 Table — (DOCX) [file pone.0298805.s008.docx]

**Table S4** Colorimetric values, including hue (H-value), saturation (S-value), and brightness (B-value), at the endpoint of DP-LAMP products with or without sunlight exposure at various viral titers (i.e., 2, 3, 4, and 5 log_10_ PFU) over time points (i.e., 0, 15, 30, and 60 min) were quantified using blue LED and orange filter and OpenCV with Python.

| Time (min) | Color value | Sunlight condition | | | | | | | | | | |  | Dark condition | | | | | | | | | | |
| --- | --- | --- | --- | --- | --- | --- | --- | --- | --- | --- | --- | --- | --- | --- | --- | --- | --- | --- | --- | --- | --- | --- | --- | --- |
|  |  | WNV Plaque-forming unit (PFU) | | | | | | | | | | |  | WNV Plaque-forming unit (PFU) | | | | | | | | | | |
|  |  | 10^5^ | |  | 10^4^ | |  | 10^3^ | |  | 10^2^ | |  | 10^5^ | |  | 10^4^ | |  | 10^3^ | |  | 10^2^ | |
|  |  | N | P |  | N | P |  | N | P |  | N | P |  | N | P |  | N | P |  | N | P |  | N | P |
| 0 | Hue | 60 | 63 |  | 61 | 58 |  | 60 | 58 |  | 58 | 58 |  | 58 | 56 |  | 60 | 57 |  | 58 | 56 |  | 59 | 57 |
|  | Saturation | 229 | 235 |  | 238 | 238 |  | 232 | 236 |  | 225 | 240 |  | 240 | 240 |  | 237 | 240 |  | 237 | 235 |  | 218 | 240 |
|  | Brightness | 79 | 94 |  | 99 | 106 |  | 90 | 120 |  | 91 | 110 |  | 73 | 91 |  | 80 | 106 |  | 84 | 96 |  | 50 | 55 |
|  |  |  |  |  |  |  |  |  |  |  |  |  |  |  |  |  |  |  |  |  |  |  |  |  |
| 15 | Hue | 58 | 56 |  | 59 | 59 |  | 59 | 57 |  | 61 | 59 |  | 59 | 60 |  | 58 | 56 |  | 61 | 57 |  | 61 | 59 |
|  | Saturation | 237 | 234 |  | 232 | 238 |  | 235 | 240 |  | 232 | 240 |  | 235 | 240 |  | 230 | 240 |  | 238 | 238 |  | 240 | 238 |
|  | Brightness | 88 | 113 |  | 80 | 110 |  | 90 | 110 |  | 85 | 111 |  | 85 | 96 |  | 91 | 94 |  | 101 | 103 |  | 96 | 102 |
|  |  |  |  |  |  |  |  |  |  |  |  |  |  |  |  |  |  |  |  |  |  |  |  |  |
| 30 | Hue | 60 | 58 |  | 59 | 58 |  | 58 | 57 |  | 58 | 57 |  | 58 | 56 |  | 58 | 58 |  | 58 | 57 |  | 59 | 58 |
|  | Saturation | 260 | 236 |  | 240 | 236 |  | 232 | 236 |  | 232 | 240 |  | 231 | 238 |  | 237 | 238 |  | 234 | 232 |  | 228 | 235 |
|  | Brightness | 93 | 102 |  | 78 | 107 |  | 81 | 107 |  | 82 | 108 |  | 76 | 91 |  | 84 | 108 |  | 78 | 113 |  | 92 | 100 |
|  |  |  |  |  |  |  |  |  |  |  |  |  |  |  |  |  |  |  |  |  |  |  |  |  |
| 60 | Hue | 64 | 60 |  | 59 | 60 |  | 59 | 58 |  | 60 | 60 |  | 64 | 59 |  | 61 | 60 |  | 61 | 60 |  | 60 | 59 |
|  | Saturation | 238 | 238 |  | 240 | 238 |  | 221 | 235 |  | 240 | 240 |  | 235 | 238 |  | 231 | 238 |  | 231 | 236 |  | 232 | 238 |
|  | Brightness | 93 | 97 |  | 75 | 97 |  | 82 | 99 |  | 71 | 96 |  | 88 | 96 |  | 73 | 104 |  | 79 | 104 |  | 82 | 94 |
